# Association of maternal dietary patterns in early pregnancy with gestational weight gain: Yazd Birth Cohort

**DOI:** 10.18502/ijrm.v23i1.18189

**Published:** 2025-03-21

**Authors:** Shahab-Aldin Akbarian, Amin Salehi-Abargouei, Sara Jambarsang, Habib Nikukar, Ph.D., Azadeh Nadjarzadeh

**Affiliations:** ^1^Department of Nutrition, School of Public Health, Shahid Sadoughi University of Medical Sciences, Yazd, Iran.; ^2^Department of Biostatistics and Epidemiology, School of Public Health, Research Center of Prevention and Epidemiology of Non-Communicable Disease, Shahid Sadoughi University of Medical Sciences, Yazd, Iran.; ^3^Medical Nanotechnology and Tissue Engineering Research Center, Yazd Reproductive Sciences Institute, Shahid Sadoughi University of Medical Sciences, Yazd, Iran.

**Keywords:** Pregnancy, Dietary patterns, Gestational weight gain.

## Abstract

**Background:**

Abnormal gestational weight gain (GWG) can carry risks for both the mother and the baby. Diet imbalances are the determining factor in the weight gain of pregnant women.

**Objective:**

This study aimed to assess the relationship between nutritional patterns and the weight of pregnant mothers living in Yazd, Iran from 2021–2022.

**Materials and Methods:**

In this cohort study, data from 1497 pregnant women aged 18–45 yr with singleton pregnancy who completed the food frequency questionnaire in the Yazd Birth Cohort Study were extracted. This data included demographic variables, GWG (difference between initial weight at 13–15 wk and 1 wk before the expected delivery date), and food intake information before the 13 ${\;}^{\text{th}}$ wk of pregnancy. The women were categorized into 3 groups based on GWG: inadequate, normal, and excessive. Dietary patterns were extracted from the food frequency questionnaire using principal component analysis, and multinomial logistic regression was used to evaluate the relationship between dietary patterns and GWG categories.

**Results:**

According to the frequency of food consumption, 3 dietary patterns were obtained: the traditional pattern (cabbage vegetables, fruits, and dried fruits), the unhealthy pattern (processed meats and sweetened drinks), and the vegetable/fruit/olive pattern. The analysis results showed that pregnant women who followed the fruit/vegetable/olive pattern had a lower chance of insufficient weight gain during pregnancy (OR: 0.66, 95% CI: 0.45–0.98).

**Conclusion:**

Consuming various fruits and vegetables can help regulate GWG in the population of pregnant women lived in Yazd, Iran. Diet can be considered one of the most effective and safe interventions.

## 1. Introduction

According to the 2021 reports of INTERGROWTH-21 ${\;}^{\text{st}}$ , 22% of pregnant women had excessive gestational weight gain (GWG), and 54% had inadequate GWG during pregnancy according to the Institute of Medicine (IOM) classification (1, 2). GWG is a complex physiological process involving adaptations for fetal and maternal tissue growth and fat deposition (3). Many recent studies have examined the relationship between maternal weight gain and fetal complications.

Excess and insufficient weight gain during pregnancy has become a major health concern in the world, which can be related to maternal diseases and delivery complications (4). Consequently, recent guidelines for pregnant women have focused more on controlling weight gain during pregnancy. Women who gain excessive weight during pregnancy (beyond recommended guidelines) have a higher risk of giving birth to babies with high birth weight, preterm delivery, and babies with a greater predisposition to childhood obesity (5). Furthermore, excessive GWG significantly increases the risk of maternal conditions such as gestational diabetes mellitus (GDM) and pre-eclampsia (6). Conversely, there is a higher likelihood of low birth weight and intrauterine growth restriction (7).

GWG is a potentially controllable risk factor because it is directly related to maternal diet. Recent research on GWG has focused on the dietary pattern of food consumption rather than on individual nutrients (8, 9) or foods (10, 11). Studying dietary patterns provides a holistic view of maternal nutrition and helps illuminate the synergistic and interactive effects of various nutrients consumed together. Although a healthy diet is essential throughout life, pregnancy requires increased nutritional intake to meet fetal demands, especially for certain nutrients. However, deficiencies in essential nutrients have been reported (12) and are linked to greater nutritional vulnerability in pregnant women (13), particularly those with poor diets. Such diets often contain more desserts, meat, meat products, sweetened drinks, and snacks, high in saturated fats, simple carbohydrates, and added sugars (14). Few studies have explored the relationship between food patterns and GWG, particularly in developing countries like Iran. Poor GWG and suboptimal maternal nutrition during pregnancy negatively impact maternal, perinatal, and fetal health outcomes (15–17).

Therefore, this study evaluates the relationship between dietary patterns extracted by the principal component analysis (PCA) method and weight gain during pregnancy.

## 2. Materials and Methods

### Study population and design

We designed a cohort study based on data from the Yazd Birth Cohort Study, which began in June 2016 and is still ongoing (18). By the time of this study, information about 3110 pregnant women living in Yazd city, Iran, who were referred to health centers before the 13 ${\;}^{\text{th}}$ wk of pregnancy and followed up at 13–15 wk, 24–27 wk, and 1 wk before delivery, was recorded in the “Yazd Birth Cohort" database.

Our inclusion criteria were pregnant women aged between 18 and 40 yr old with a singleton pregnancy who completed the food frequency questionnaire (FFQ). Women who smoked, consumed alcohol, had incomplete maternal weight data, chronic diseases, or energy intake below 800 or above 4000 kcal/day according to their FFQ analysis were excluded from the study.

In the main cohort study, mothers who referred to health centers before the 13 ${\;}^{\text{th}}$ wk of pregnancy were recruited to the study. Demographic and FFQ questionnaires were conducted during initial face-to-face interviews, with follow-ups scheduled at 3 intervals: 13–15 wk, 24–27 wk, and 1 wk before the expected delivery date.

### Dietary assessment

In this study, we utilized dietary information recorded in the FFQ completed by pregnant women participating in the main cohort study completed at the first visit ( < 13 wk). This was an 88-item food questionnaire and its validity and reliability had already been investigated. Sharifi and colleagues obtained a Pearson correlation coefficient of r = 0.845 between test and retest for foods (18).

Additionally, tests of sampling adequacy showed sufficiency (Kaiser-Meyer-Olkin was 0.195, and the p-values for the Bartlett test of sphericity were all less than 0.001). For each food item in the FFQ, women were asked to report the frequency of consumption (daily, weekly, monthly, or yearly) for defined portion sizes (standard units commonly used by Iranians). Participants reported consumption frequency for each item based on standard Iranian portion sizes. Daily dietary intake (gr) was calculated from frequency data, and nutrient and energy intakes were derived using the Nutritionist 4 software.

### Dietary pattern

Factor analysis, specifically PCA, was employed to identify posteriori dietary patterns. 88 foods were grouped based on nutrient similarity and culinary use (34 non-overlapping groups, Table I). Food items were retained individually if they constituted a single food item (e.g., eggs, canned fish, tomatoes, etc.) or if they were a specific feature of a dietary pattern (e.g., chips and salt). To eliminate the possible confounding effects of energy, food group consumption was standardized to a 1000 kcal basis; then energy-adjusted values were entered into PCA analysis which rotated by orthogonal transformation (varimax rotation). The number of dietary patterns retained was based on the inflection point in the scree plot and the interpretability of the factors. Factor loadings indicate the strength and direction of the relationship between factors (dietary patterns) and food groups. A positive factor loading indicates greater consumption of that food group in the same pattern, and a negative factor loading indicates lesser consumption. Finally, each participant receives a factor score for each dietary pattern, calculated using multiple regression. These scores indicate the correspondence of participants' dietary patterns with the identified dietary patterns. For each dietary pattern, individuals are ranked into quartiles (quartile 1 represented a low consumption of the dietary pattern while quartile 4 represented a high consumption).

### GWG and other covariates 

Data on participants' weight and height were extracted from the Yazd Birth Cohort. Body mass index (BMI) was calculated by dividing weight in kilograms by the square of height in meters. Weight during the second visit (13–15 wk) was used as the initial gestational weight. The mothers' final weight was the one measured at the visit 1 wk before their estimated delivery date. The difference between the initial weight and 1 wk before the expected delivery date was used to calculate GWG. The GWG of pregnant mothers was classified according to IOM guidelines: 12.5–18 kg for underweight, 11.5–16 kg for normal weight, 7–11.5 kg for overweight, and 5–9 kg for obese women. Weight gain less than or greater than the IOM recommended range was classified as inadequate or excessive GWG.

Potential confounding factors, such as maternal age, BMI, and height, were considered as continuous variables. Physical activity data, obtained using the international physical activity questionnaire, was divided into 3 categories: low ( < 600 MET min/wk), moderate (600–3000 MET min/wk), and high ( > 3000 MET min/wk). Additional data, such as the use of oral contraceptive pills in the 4 months before pregnancy, diabetes, primiparity, and maternal education, were extracted from the main cohort database. Maternal education was categorized into 3 groups: illiterate, poorly educated, and university educated (Table II).

**Table 1:** Factor loading of energy-adjusted food groups in 3 main dietary patterns

**Food groups**	**Items**	**Traditional pattern**	**Unhealthy pattern**	**Vegetable/fruit/olive pattern**
**Cruciferous vegetables**	All types of cruciferous vegetable	0.73	-	-
**Leafy vegetables**	Stew herbs, leafy greens, lettuce	0.64	-	0.21
**Other vegetables**	Cucumber, zucchini, eggplant, celery, mushrooms, carrots, bell peppers	0.56	-	0.4
**Processed meat**	All types of sausages and hot dogs	-	0.72	-
**Soft drinks**	Nonalcoholic beer and soft drinks	-	0.68	-
**Salt**	Salt	-	0.28	-
**Sulfuric vegetables**	Garlic and onion	-	-	0.63
**Tomato**	Tomato	0.25	-	0.59
**Fruits**	Apple, banana, citrus, grape, cantaloupe, melon, watermelon, pear, apricot, nectar, peach, cherry, sour cherry, kiwi, pomegranate, plum, white mulberry	0.23	-	0.45
**Olive**	Olive	-	-	0.38
**Dried fruits**	Dried white mulberry, dried fig, dried date, and all kinds of dried fruits	0.28	-	-
**Canned fish**	Canned fish	-	0.21	-
**Chips and puffs**	All kinds of chips and puffs	-	0.22	-0.21
**Dairy**	Milk, cheese, yogurt, buttermilk, curd, ice cream	0.2	-	-
**Egg**	Egg	-	-.22	-
**Date**	Date	0.2	-0.26	-
**Pickles**	All types of pickles	0.3	0.25	-
Values are factor loading (correlation coefficients) between factors (dietary patterns) and food frequency variables. Foods are sorted by size of loading factors, and minus values show a lower frequency of foods in the pattern. Absolute values < 0.20 are removed to simplify				

**Table 2:** Baseline characteristics of pregnant women across weight gain categories

**Variables **		**Inadequate weight gain (n = 411)**	**Normal weight gain (n = 588)**	**Excessive weight gain (n = 498)**	**P-value**
Age (yr)*		28 (7)	29 (8)	28 (7)	0.63
BMI (kg/m^2^)*		23.71 (5.25)	25.05 (5.83)	26.67 (5.34)	< 0.001
Height (cm)*		159 (7)	158.7 (7.5)	160.5 (8.3)	0.04
BMI category**					
	**< 18.5**	38 (9.2)	29 (4.9)	9 (1.8)	< 0.001
	**18.5–25**	249 (60.6)	262 (44.6)	128 (25.7)	
	**25–30**	93 (22.6)	218 (37.1)	252 (50.6)	
	**> 30**	31 (7.5)	79 (13.4)	109 (21.9)	
Education**					
	**Illiterate**	220 (53.5)	281 (47.8)	233 (46.8)	0.04
	**Low literacy**	168 (40.9)	271 (46.1)	218 (43.8)	
	**University**	23 (5.6)	36 (6.1)	47 (9.4)	
Physical activity**					
	**Low**	224 (60.2)	347 (63.8)	298 (64.6)	0.45
	**Medium**	135 (36.3)	171 (31.4)	145 (31.5)	
	**High**	13 (3.5)	26 (4.8)	18 (3.9)	
Nulliparous**		282 (68.8)	397 (68)	340 (68.3)	0.96
GDM**		65 (17.6)	72 (13.1)	57 (12.5)	0.07
Previous pregnancy GDM**		20 (4.9)	26 (4.4)	21 (4.2)	0.89
OCP used**		43 (10.5)	57 (9.7)	63 (12.7)	0.29
*Data presented as median and interquartile range (IQR), ANOVA test. **Data presented as n (%), Chi-square test. BMI: Body mass index, GDM: Gestational diabetes mellitus, OCP: Oral contraceptive pills					

### Ethical Considerations

The study protocol was approved by the ethics committee of School of Public Health, Shahid Sadoughi University of Medical Sciences, Yazd, Iran (Code: IR.SSU.SPH.REC.1399.201), granting permission to access the study data. All personal information is kept confidential and will be used anonymously for scientific purposes only.

### Statistical Analysis

Qualitative variables are presented as numbers and percentages, and quantitative variables are presented as mean and standard deviation. Quantitate characters of the population (age, BMI, and height) reported as median and interquartile range. Before starting the analyses, the normal distribution of the variables was performed using the Kolmogorov-Smirnov method, and continuous variables having a skewed distribution were also tested using the Kruskal-Wallis test. The Pearson Chi-square test was employed for qualitative variables, and the analysis of variance (ANOVA) test was used to assess differences in the means distribution. Basic maternal characteristics were evaluated across 3 BMI-specific GWG categories defined by the IOM: inadequate, normal, and excessive weight gain. Macro and micronutrients are reported in table III according to BMI-specific GWG categories, which are adjusted for energy, calculated by the analysis of covariance (ANCOVA) test.

PCA with varimax rotation was utilized to identify dietary patterns. The Kaiser-Meyer-Olkin measure of sampling adequacy (0.45) and Bartlett's test of sphericity (p < 0.001) confirmed the appropriateness of PCA for this study. Food groups with factor loadings > 0.02 were strongly associated with a dietary pattern. Also, negative loading factors represent lower consumption of that food group. Since both inadequate and excessive GWG are independent disorders, we used the multinomial regression analysis method, which considered normal GWG as a reference, and both inadequate and excessive GWG- were compared with normal GWG.

## 3. Results

### Characteristics of population

**Figure 1:**
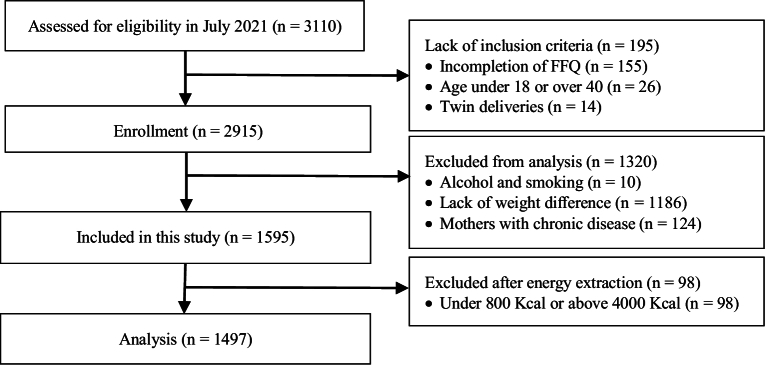
Flow chart of study population. FFQ: Food frequency questionnaire.

A total of 3114 pregnant women were registered in the Yazd Birth Cohort Study database up to the time of this study. Based on our inclusion criteria, 2915 women were initially included in the study. Participants were excluded in the first stage due to smoking and alcohol consumption (n = 10), no recorded weight difference (1186 women), and chronic diseases such as diabetes (excluding GDM), cancers, fibromyalgia, chlamydia, rheumatoid arthritis, systemic lupus erythematosus, autoimmune diseases, syphilis, gonorrhea, toxoplasmosis, epilepsy, and multiple sclerosis (n = 124). In the next stage, after extracting information and calculating the energy intake of the participants based on their FFQ, 98 women whose energy intake was less than 800 or more than 4000 kcal per day were also excluded from the study. Finally, the data of 1497 pregnant women were analyzed (Figure 1).

Based on pre-pregnancy BMI, the distribution of women was as follows: underweight: 76 (5.1%), normal weight: 639 (42.7%), overweight: 563 (37.6%), and obese: 219 (14.6%). Out of 1497 pregnant women, 588 mothers had normal GWG, 411 mothers had inadequate GWG, and 498 mothers had excessive GWG. Sociodemographic, anthropometric, and lifestyle data are mentioned in detail in table II, based on maternal GWG categories. Although maternal age did not differ significantly across groups, women in the excessive GWG group had higher initial BMI and height compared to the normal GWG group, with the normal group surpassing the inadequate GWG group. A higher number of mothers with university education appeared in the excessive GWG category, while those with less education were more likely to fall into the normal GWG category.

### Micro and macronutrients and weight gain category

Table III shows the average macro and micronutrients in GWG categories. The total energy intake did not significantly differ between inadequate GWG, normal GWG, and excessive GWG groups. Among all macronutrients and micronutrients, protein intake was the only one with a higher mean in the excessive GWG group (p = 0.01), while all items were adjusted for energy intake.

### Dietary patterns

**Figure 2:**
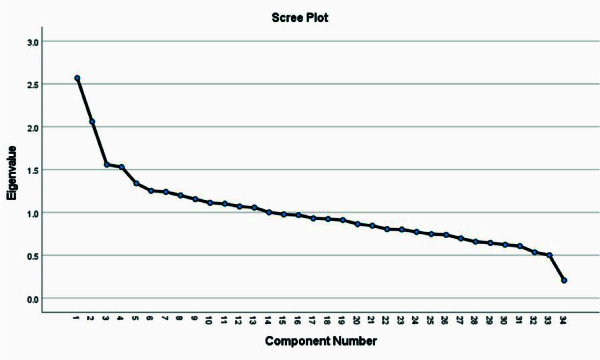
The scree plot shows the inflection point after third factor.

14 dietary patterns were identified with eigenvalues above one. Based on the breakpoint in the scree plot and interpretability, 3 main factors were selected (Figure 2), accounting for 18.1% of the total variance in food intake. The first factor, “traditional”, aligned closely with the typical Iranian diet, including cruciferous and leafy vegetables, other vegetables, tomatoes, fruits, dried fruits, dairy products, dates, and pickles. The second pattern, “unhealthy”, comprised higher intakes of processed meats, soft drinks, salt, canned fish, chips and puffs, pickles, and lower intakes of eggs and dates. The third pattern, “vegetable/fruit/olive”, was characterized by higher intakes of sulfuric vegetables, tomatoes, fruits, other vegetables, olives, and leafy vegetables, with lower chips and puffs. Factor loadings below the absolute value of 0.2 are not shown in the table.

### Dietary patterns and GWG

Table IV presents the crude and adjusted models of the relationship between dietary patterns and GWG. No significant associations were observed between traditional and unhealthy dietary patterns with inadequate or excessive GWG. However, for the “vegetable/fruit/olive” pattern, a significant inverse relationship was found with inadequate GWG. This was significant in the adjusted model between the 2 ${\;}^{\text{nd}}$ and 4 ${\;}^{\text{th}}$ quartiles compared to the 1 ${\;}^{\text{st}}$ quartile (OR: 0.54; 95% CI: 0.37–0.79 and OR: 0.66; 95% CI: 0.45–0.98, respectively).

**Table 3:** Macro and micronutrients intakes in study population at early pregnancy

**Variables **	**Inadequate weight gain (n = 411)**	**Normal weight gain (n = 588)**	**Excessive weight gain (n = 498)**	**P-value**
Energy*	2287.1 ± 627	2281.9 ± 641	2320.2 ± 611	0.57
Carbohydrate**	328.9 ± 1.85	323.9 ± 1.55	325.9 ± 1.68	0.11
Protein**	69.38 ± 0.55	69.34 ± 0.46	71.18 ± 0.5	0.01
Fat**	81.4 ± 0.84	83.4 ± 0.71	81.9 ± 0.77	0.15
Saturated fat**	24.57 ± 0.4	24.87 ± 0.33	24.8 ± 0.36	0.84
Poly-unsaturated fat**	21.81 ± 0.49	22.48 ± 0.41	22.04 ± 0.44	0.55
Sugar**	91.14 ± 1.7	91.89 ± 1.4	94.77 ± 1.6	0.25
Total fiber**	19.78 ± 0.32	19.52 ± 0.27	20.55 ± 0.29	0.3
Zinc**	7.69 ± 0.08	7.8 ± 0.07	8.01 ± 0.7	0.2
Vitamin C**	117.6 ± 3.32	118.4 ± 2.78	123.3 ± 3.02	0.36
Iron**	9.26 ± 0.19	9.28 ± 0.09	9.41 ± 0.09	0.54
*Data presented as Mean ± SD, ANOVA test. **Data presented as Mean ± SE, ANCOVA test, adjusted for energy intake				

**Table 4:** Association between dietary patterns quartiles and inadequate and excessive GWG in study population

		**Crude model**				**Model 1**			
**Variables**		**Inadequate weight gain**		**Excessive weight gain**		**Inadequate weight gain**		**Excessive weight gain**	
		**OR (95% CI)**	**P-value**	**OR (95% CI)**	**P-value**	**OR (95% CI)**	**P-value**	**OR (95% CI)**	**P-value**
**Traditional pattern**									
	**Q 1**	1 Ref.		1 Ref.		1 Ref.		1 Ref.	
	**Q 2**	0.77 (0.54–1.11)	0.16	1.31 (0.93–1.86)	0.11	0.78 (0.53–1.15)	0.21	1.28 (0.88–1.84)	0.18
**Traditional pattern**									
	**Q 3**	0.99 (0.69–1.4)	0.95	1.4 (0.99–1.99)	0.05	1.01 (0.69–1.48)	0.93	1.35 (0.93–1.97)	0.11
	**Q 4**	0.7 (0.49–1.01)	0.05	1.27 (0.9–1.8)	0.16	0.68 (0.46–1.02)	0.06	1.14 (0.78–1.65)	0.48
**Unhealthy pattern**									
	**Q 1**	1 Ref.		1 Ref.		1 Ref.		1 Ref.	
	**Q 2**	1.17 (0.82–1.68)	0.36	0.87 (0.62–1.22)	0.43	1.22 (0.82–1.8)	0.31	0.89 (0.62–1.29)	0.56
	**Q 3**	0.94 (0.65–1.36)	0.76	0.85 (0.61–1.2)	0.36	0.95 (0.64–1.41)	0.8	0.85 (0.59–1.22)	0.39
	**Q 4**	0.97 (0.67–1.4)	0.9	0.94 (0.67–1.32)	0.74	0.89 (0.59–1.33)	0.57	0.83 (0.58–1.21)	0.35
**Vegetable/fruit/olive pattern**									
	**Q 1**	1 Ref.		1 Ref.		1 Ref.		1 Ref.	
	**Q 2**	0.54 (0.38–0.78)	0.001*	0.83 (0.59–1.18)	0.31	0.54 (0.37–0.79)	0.002*	0.87 (0.6–1.25)	0.46
	**Q 3**	0.75 (0.53–1.07)	0.12	1.04 (0.73–1.47)	0.8	0.71 (0.48–1.04)	0.08	1.04 (0.72–1.5)	0.82
	**Q 4**	0.77 (0.53–1.09)	0.15	1.07 (0.76–1.52)	0.67	0.66 (0.45–0.98)	0.04*	1.04 (0.71–1.5)	0.83
Q: Quartile, Ref: Reference, OR: Odds ratio, CI: Confidence interval, results from multinomial regression model. Q 1 is considered as a reference. Model 1: Adjusted for gestational diabetic mellitus in a previous pregnancy, education level, physical activity, age, used oral contraceptive pill duration 4 wk before this pregnancy, infant gender, nulliparous, gestational diabetic mellitus									

## 4. Discussion 

To our knowledge, the present study is the first to measure the relationship between dietary patterns and weight gain of pregnant women in a large prospective cohort study in an urban population in Iran. 3 distinct dietary patterns were extracted in this study: traditional, unhealthy, and vegetables/fruits/olive patterns. Our findings suggest that higher consumption of the vegetable/fruit/olive dietary pattern is associated with a reduced likelihood of inadequate GWG.

Dietary intervention is one of the safest and most effective strategies often overlooked in pregnancy. Recent nutritional epidemiological studies have tended to focus on individual micronutrients or macronutrients rather than evaluating comprehensive dietary patterns (19). In addition, assessing food patterns provides a broader and more practical perspective that can better inform healthy eating recommendations (20).

While some studies have reported a positive association between unhealthy dietary patterns and excessive GWG in pregnant women (21, 22), we observed no such association in our study. This finding contrasts with previous research indicating that diets high in energy density are linked to excessive GWG among European women (23). One possible explanation for this discrepancy is that pregnant women who gain excessive weight are often already overweight or obese and may under-report their consumption of high-calorie foods. Consequently, they may over-report healthy food intake, introducing bias into the results, as discussed in other studies (5, 24).

Our study indicated that a dietary pattern rich in fruits, vegetables, and olives correlates with a decreased risk of inadequate GWG despite some studies failing to find such a relationship (25, 26). For example, the Norwegian Mother and Child Cohort Study found that women with normal weight adhering to the New Nordic Diet, which is high in fruits and vegetables, had a lower risk of excessive GWG (27). Similarly, Zhang and colleagues analyzed 833 pregnant women and identified that total energy intake and consumption of sweet beverages were positively associated with excessive GWG (28). Another Iranian study of 488 pregnant women found 2 dietary patterns: high-fat/fast-food and fruit/vegetable/protein patterns. Their results indicated that the high-fat/fast-food pattern increased the risk of GWG and high blood sugar, whereas the fruit/vegetable/protein pattern was linked to a reduced risk of high blood sugar (29). A distinguishing feature of this study is that significant results were observed in the third trimester, associated with higher total energy and carbohydrate intake compared to earlier trimesters (30).

In line with our findings, a study from the UAE identified 2 dietary patterns: A western pattern characterized by sweets, sugar-sweetened beverages, and fast foods and a diverse pattern including fruits, vegetables, meat, dairy, grains, legumes, and nuts. Participants in the highest quartile of the diverse pattern exhibited a significantly lower risk of inadequate GWG compared to the lowest quartile. In contrast, those in the highest quartile of the western pattern had a four-fold increase in the risk of excessive GWG (16).

The inconsistencies in the relationship between GWG and dietary patterns across studies may stem from the differing compositions of dietary patterns analyzed. In our study, reducing the chance of insufficient GWG can be justified by the fact that the variety of food groups in the traditional food pattern provides a balance in the intake of energy, macro, and micronutrients. At the same time, it has no effect on the chance of excessive GWG (31). Typical foods in the traditional pattern (fruits and vegetables) are rich sources of fiber, vitamins, minerals, and antioxidants that have protective effects on the immune system, antioxidant defense, and normal regulation of hormonal metabolism (32). Low energy density and high micronutrient content of fruits and vegetables help maintain a healthy weight during pregnancy. Additionally, individuals with higher fruit and vegetable intake may lead healthier lifestyles, potentially increasing the likelihood of staying within the normal GWG range (33). Further research is essential to explore the underlying mechanisms linking these food groups with GWG.

The strengths of this study include its prospective design and large sample size. It is one of the first population-based investigations in Iran to evaluate dietary habits alongside GWG and various confounders. However, limitations exist.

The limited number of food items in the FFQ may not accurately represent total energy and nutrient intake (33). GWG was calculated based on the weight difference between 13 and 15 wk and just before delivery, which might introduce error since early pregnancy weight can differ from that measured at 13–15 wk. Moreover, while multiple assessments of maternal weight (monthly or at least 5 times) allowed for examining diet's impact on GWG, future studies should also consider the composition of GWG, including body water, fat mass, and fat-free mass.

## 5. Conclusion

The present study identified 3 dietary patterns among pregnant women lived in Yazd, Iran: 1) Traditional, 2) Unhealthy, and 3) vegetable/fruit/olive. The vegetable/fruit/olive pattern demonstrated an inverse relationship with the risk of inadequate weight gain, while the traditional and unhealthy patterns did not yield significant results. These findings underscore the importance of consuming fruits and vegetables, which may play a crucial role in weight regulation during pregnancy.

##  Data Availability

Data will be made available on request from the corresponding author.

##  Author Contributions

Sh-A. Akbarian: Conceptualization, design of the work and drafting the work. A. Nadjarzadeh: Design of the work, data curation, project administration, interpretation of data for the work, and manuscript revising. A. Salehi-Abargouei: Data curation, manuscript revising and supervision. S. Jambarsang: Data curation, manuscript revising, analysis and interpretation of data for the work. H. Nikukar: Acquisition and manuscript revising. We all contributed to and approved the final manuscript.

##  Conflict of Interest 

The authors declare that there is no conflict of interest.

## References

[bib1] Goldstein RF, Abell SK, Ranasinha S, Misso M, Boyle JA, Black MH, et al (2017). Association of gestational weight gain with maternal and infant outcomes: A systematic review and meta-analysis. JAMA.

[bib2] Darling AM, Wang D, Perumal N, Liu E, Wang M, Ahmed T, et al (2023). Risk factors for inadequate and excessive gestational weight gain in 25 low-and middle-income countries: An individual-level participant meta-analysis. PLoS Med.

[bib3] Champion ML, Harper LM (2020). Gestational weight gain: Update on outcomes and interventions. Curr Diab Rep.

[bib4] Li M, Zhang C-Y, Yue C-Y (2022). Effects of pre-pregnancy BMI and gestational weight gain on adverse pregnancy outcomes and complications of GDM. J Obstet Gynaecol.

[bib5] Cano-Ibáñez N, Martínez-Galiano JM, Luque-Fernández MA, Martín-Peláez S, Bueno-Cavanillas A, Delgado-Rodríguez M (2020). Maternal dietary patterns during pregnancy and their association with gestational weight gain and nutrient adequacy. Int J Environ Res Public Health.

[bib6] Gilmore LA, Klempel-Donchenko M, Redman LM (2015). Pregnancy as a window to future health: Excessive gestational weight gain and obesity. Semin Perinatol.

[bib7] Guan P, Tang F, Sun G, Ren W (2019). Effect of maternal weight gain according to the Institute of Medicine recommendations on pregnancy outcomes in a Chinese population. J Int Med Res.

[bib8] Tebbani F, Oulamara H, Agli A (2019). [Factors associated with low maternal weight gain during pregnancy]. Rev Epidemiol Sante Publique.

[bib9] Campos CAS, Malta MB, Neves PAR, Lourenço BH, Castro MC, Cardoso MA (2019). Gestational weight gain, nutritional status and blood pressure in pregnant women. Rev Saude Publica.

[bib10] Tovar A, Kaar JL, McCurdy K, Field AE, Dabelea D, Vadiveloo M (2019). Maternal vegetable intake during and after pregnancy. BMC Pregnancy Childbirth.

[bib11] Lai JS, Soh SE, Loy SL, Colega M, Kramer MS, Chan JKY, et al (2019). Macronutrient composition and food groups associated with gestational weight gain: The GUSTO study. Eur J Nutr.

[bib12] Gernand AD, Schulze KJ, Stewart CP, West Jr KP, Christian P (2016). Micronutrient deficiencies in pregnancy worldwide: Health effects and prevention. Nat Rev Endocrinol.

[bib13] Cano-Ibáñez N, Martínez-Galiano JM, Amezcua-Prieto C, Olmedo-Requena R, Bueno-Cavanillas A, Delgado-Rodríguez M (2020). Maternal dietary diversity and risk of small for gestational age newborn: Findings from a case-control study. Clin Nutr.

[bib14] Martínez-González MÁ, Martín-Calvo N (2013). The major European dietary patterns and metabolic syndrome. Rev Endocr Metab Disord.

[bib15] Plante AS, Lemieux S, Labrecque M, Morisset AS (2019). Relationship between psychosocial factors, dietary intake and gestational weight gain: A narrative review. J Obstet Gynaecol Can.

[bib16] Itani L, Radwan H, Hashim M, Hasan H, Obaid RS, Ghazal HA, et al (2020). Dietary patterns and their associations with gestational weight gain in the United Arab Emirates: Results from the MISC cohort. Nutr J.

[bib17] Saldiva SRDM, De Arruda Neta AdCP, Teixeira JA, Peres SV, Marchioni DML, Carvalho MA, et al (2022). Dietary pattern influences gestational weight gain: Results from the ProcriAr cohort study-São Paulo, Brazil. Nutrients.

[bib18] Sharifi SF, Javadi M, Barikani A (2016). Reliability and validity of short food frequency questionnaire among pregnant females. Biotechnol Health Sci.

[bib19] Tielemans MJ, Garcia AH, Santos AP, Bramer WM, Luksa N, Luvizotto MJ, et al (2016). Macronutrient composition and gestational weight gain: A systematic review. Am J Clin Nutr.

[bib20] Schulz Ch-A, Oluwagbemigun K, Nöthlings U (2021). Advances in dietary pattern analysis in nutritional epidemiology. Eur J Nutr.

[bib21] Tielemans MJ, Erler NS, Leermakers ET, Van den Broek M, Jaddoe VW, Steegers EA, et al (2015). A priori and a posteriori dietary patterns during pregnancy and gestational weight gain: The generation R study. Nutrients.

[bib22] Guilloty NI, Soto R, Anzalota L, Rosario Z, Cordero JF, Palacios C (2015). Diet, pre-pregnancy BMI, and gestational weight gain in Puerto Rican women. Matern Child Health J.

[bib23] Maugeri A, Barchitta M, Favara G, La Rosa MC, La Mastra C, Magnano San Lio R, et al (2019). Maternal dietary patterns are associated with pre-pregnancy body mass index and gestational weight gain: Results from the “mamma & bambino” cohort. Nutrients.

[bib24] Wrottesley SV, Pisa PT, Norris SA (2017). The influence of maternal dietary patterns on body mass index and gestational weight gain in urban black South African women. Nutrients.

[bib25] Ferreira LB, Lobo CV, Miranda AEdS, Carvalho BdC, Santos LCd (2022). Dietary patterns during pregnancy and gestational weight gain: A systematic review. Rev Bras Ginecol Obstet.

[bib26] Shin D, Lee KW, Song WO (2016). Pre-pregnancy weight status is associated with diet quality and nutritional biomarkers during pregnancy. Nutrients.

[bib27] Hillesund ER, Bere E, Haugen M, Øverby NC (2014). Development of a New Nordic Diet score and its association with gestational weight gain and fetal growth-a study performed in the Norwegian Mother and Child Cohort Study (MoBa). Public Health Nutr.

[bib28] Zhang S, Zhang C, Guo J, Li B, Li W, Liu J, et al (2024). Higher sweet beverage consumption was associated with increased gestational weight gain and birth weight: A Chinese cohort study. Nutr Res.

[bib29] Angali KA, Shahri P, Borazjani F (2020). Maternal dietary pattern in early pregnancy is associated with gestational weight gain and hyperglycemia: A cohort study in South West of Iran. Diabetes Metab Syndr.

[bib30] Tayyem R, Allehdan SS, Al-Awwad NJ, Alatrash RM, Mahfouz IA, Alasali F (2020). Food group intake of pregnant jordanian women based on the three pregnancy trimesters. Prev Nutr Food Sci.

[bib31] Zhou M, Peng X, Yi H, Tang S, You H (2022). Determinants of excessive gestational weight gain: A systematic review and meta-analysis. Arch Public Health.

[bib32] Jideani AI, Silungwe H, Takalani T, Omolola AO, Udeh HO, Anyasi TA (2021). Antioxidant-rich natural fruit and vegetable products and human health. Int J Food Propert.

[bib33] Wei X, He J-R, Lin Y, Lu M, Zhou Q, Li S, et al (2019). The influence of maternal dietary patterns on gestational weight gain: A large prospective cohort study in China. Nutrition.

